# Supplementary material for: Nearest-neighbour resonating valence bonds in YbMgGaO4
Source: Nat Commun. 2017 Jun 22;8:15814. doi: 10.1038/ncomms15814 (PMC5489678; doi:10.1038/ncomms15814)
Supplement: Supplementary Information — Supplementary Notes, Supplementary Figures, Supplementary Table and Supplementary References [file ncomms15814-s1.pdf]

Supplementary Note 1. Diagonalization calculation for the Yb^{3+} dimer in $YbMgGaO_4$

The crystal field ground state of Yb^{3+} in $YbMgGaO_4$ has been studied in our recent high-energy INS work[1]. The wavefunctions of the ground-state Kramers doublet are ~ ±0.71|±7/2>∓0.36|∓5/2>+0.60|±1/2>. The effective spin-1/2 *g*-factors ($g_{||}$ = 3.72 and $g_{\perp}$ = 3.06) had also been reported in our previously reported work[2].

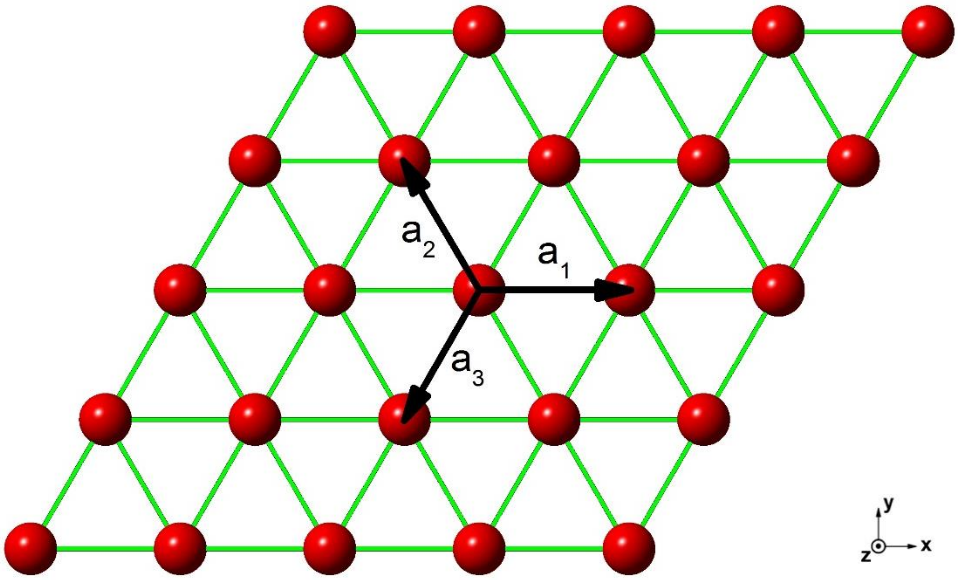

Supplementary Figure 1. Triangular lattice of Yb^{3+} in $YbMgGaO_4$. The red spheres represent the Yb^{3+} ions, and the bonds along the $\mathbf{a}_1$, $\mathbf{a}_2$, $\mathbf{a}_3$ directions are labeled.

The effective spin-1/2 Hamiltonian for the Yb^{3+} dimer ($\mathbf{S}_1$ and $\mathbf{S}_2$) had been reported in our previous work[2].

$$\mathbf{H} = J_{zz}S_1^zS_2^z + J_{\pm}(S_1^+S_2^- + S_1^-S_2^+) + J_{\pm\pm}(\gamma_{12}S_1^+S_2^+ + \gamma_{12}{}^*S_1^-S_2^-) - \frac{iJ_{z\pm}}{2}(\gamma_{12}{}^*S_1^+S_2^z - \gamma_{12}S_1^-S_2^z + < 1 \leftrightarrow 2 >). \qquad (1)$$

Here, $J_{\pm}$ ~ 0.9 K, J_{zz} ~ 0.98 K, $J_{z\pm}$ ~ 0 K, $J_{\pm\pm}$ ~ (+ or -)0.155 K, and γ_{12} = 1, $e^{i2\pi/3}$, $e^{-i2\pi/3}$ for the bonds along the $\mathbf{a}_1$, $\mathbf{a}_2$, $\mathbf{a}_3$ directions (Supplementary Fig. 1), respectively.

Through the diagonalization of the Hamiltonian (Supplementary Eq. 1), we find that the ground state is the ***strict*** traditional singlet, $\frac{1}{\sqrt{2}}(|\uparrow\downarrow> - |\downarrow\uparrow>)$. It is independent on the bond direction ($\mathbf{a}_1$, $\mathbf{a}_2$, or $\mathbf{a}_3$) and the anisotropic coupling parameters, as long as the isotropic coupling, $J_0 \equiv (4J_{\pm}+J_{zz})/3$, is antiferromagnetic (i.e. $J_0 > 0$). And J_0 = 1.5(1) K or 0.13(1) meV > 0 had been safely determined in our previous work[2].

The energy separations between the ground-state singlet (with the eigenvalue of energy, $-3/4J_0$) and three excited states are also independent of the bond direction ($\mathbf{a}_1$, $\mathbf{a}_2$, or $\mathbf{a}_3$), but are strongly dependent on the anisotropic coupling parameters. Since $J_{z\pm}$ had been measured to be almost zero[2], all of the three excited energies (from $-3/4J_0$) are independent on the sign of $J_{\pm\pm}$ at $J_{z\pm}$ = 0, and are calculated to be $0.809J_0$, $1.012J_0$, and $1.179J_0$, respectively.

As a result, the uncorrelated spin-1/2 nearest-neighbor valence bond model, which had been used for herbertsmithite[3], is applicable to $YbMgGaO_4$ as well.

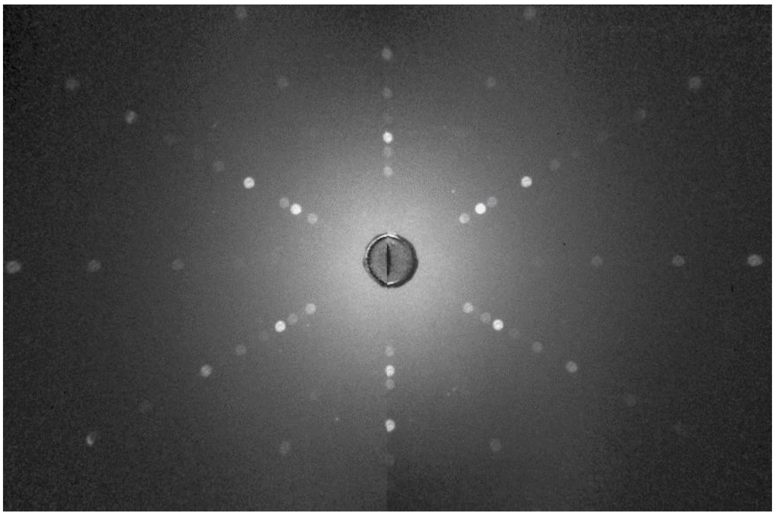

Supplementary Figure 2. Laue X-ray diffraction. The pattern was collected for the (0, 0, 1) surface of the $YbMgGaO_4$ single crystal.

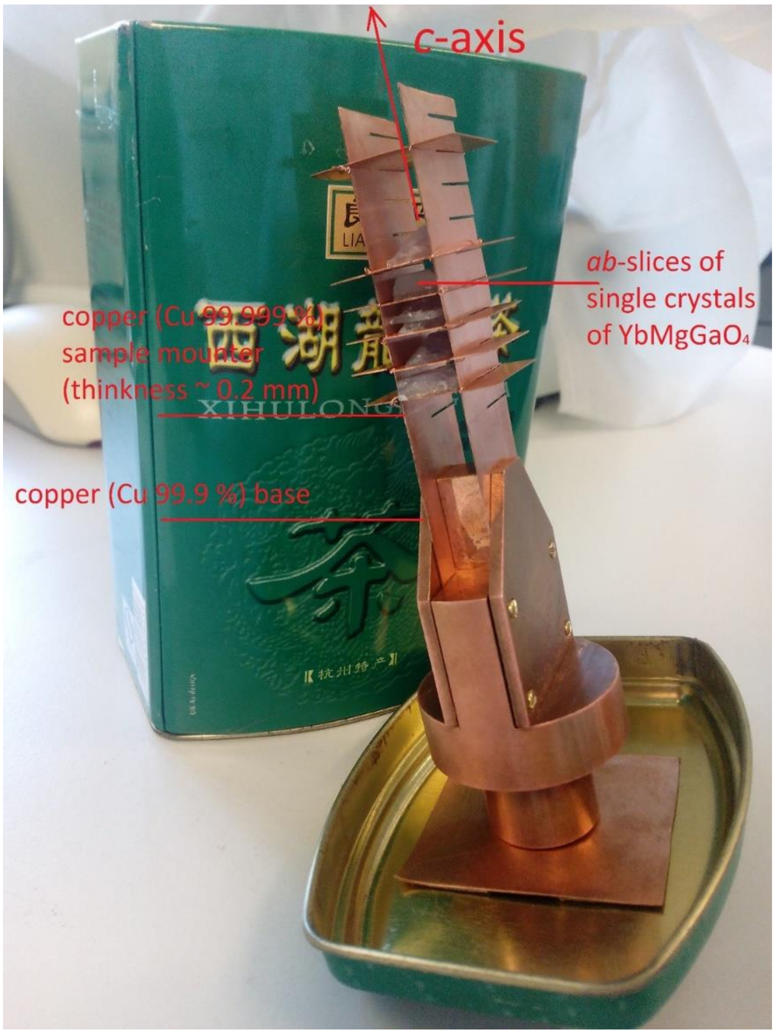

Supplementary Figure 3. $YbMgGaO_4$ single-crystal sample. The sample (including a self-made sample mounter) was used in the neutron scattering measurements.

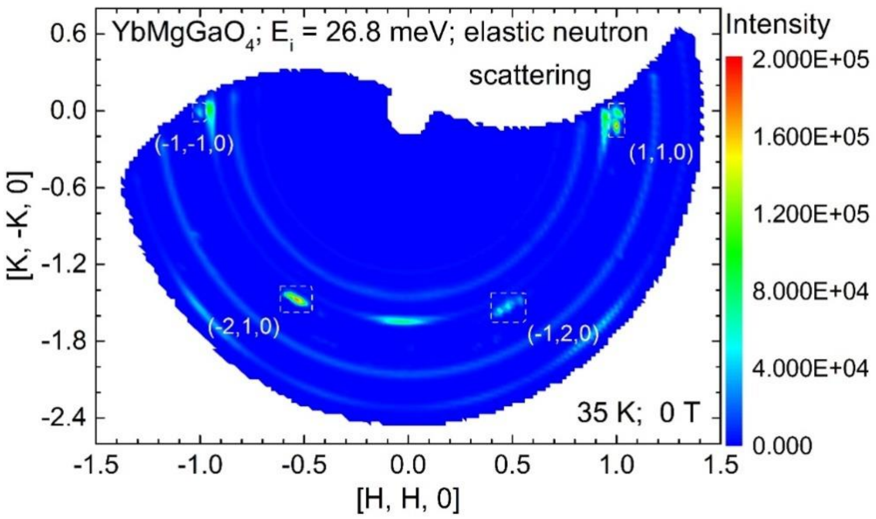

Supplementary Figure 4. Neutron diffraction. The pattern for the $YbMgGaO_4$ single-crystal sample was collected at 35 K and 0 T (E_i = 26.8 meV).

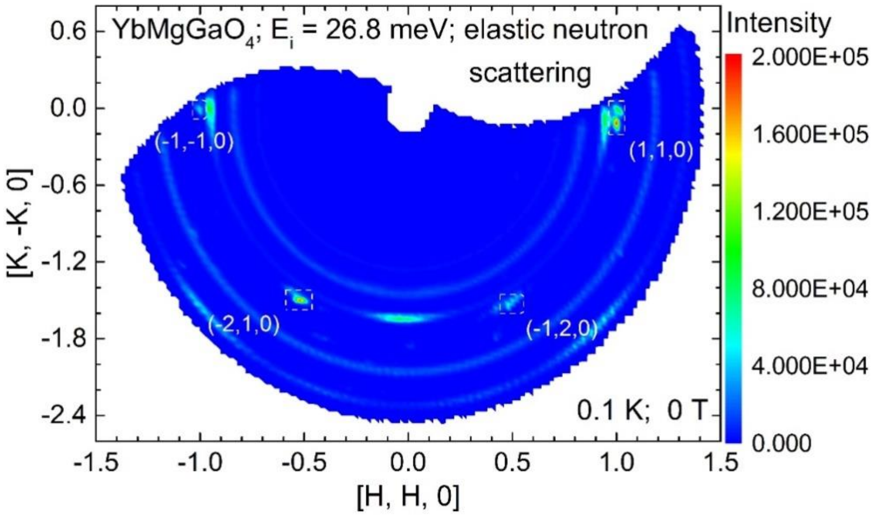

Supplementary Figure 5. Neutron diffraction. The pattern for the $YbMgGaO_4$ single-crystal sample was collected at 0.1 K and 0 T (E_i = 26.8 meV).

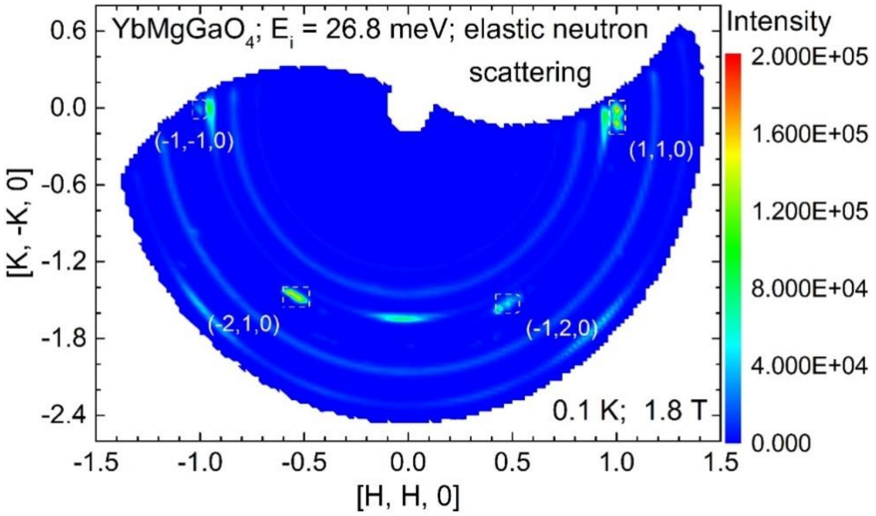

Supplementary Figure 6. Neutron diffraction. The pattern for the $YbMgGaO_4$ single-crystal sample was collected at 0.1 K and 1.8 T (E_i = 26.8 meV).

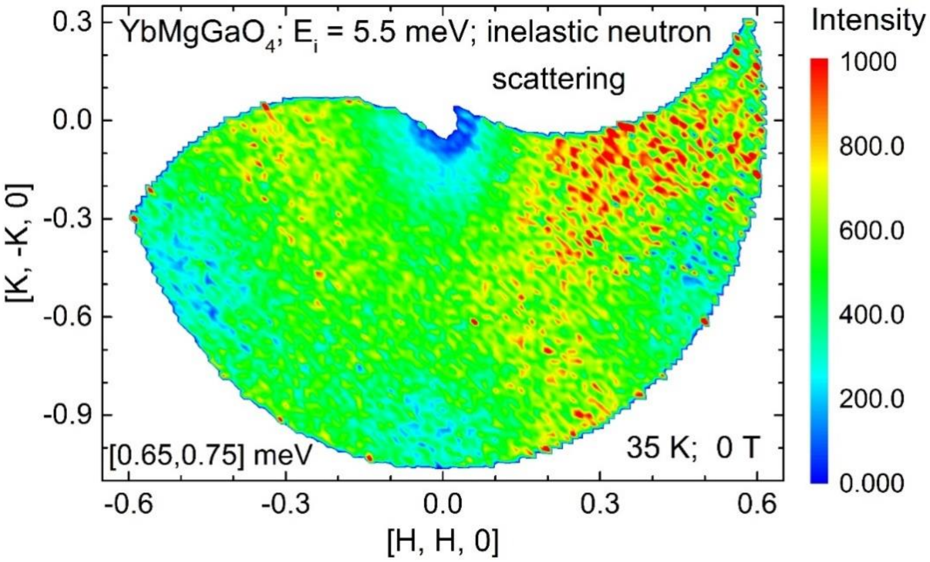

Supplementary Figure 7. Raw inelastic neutron scattering spectrum. The data have been integrated over the momentum space, $-0.9 \leq \eta \leq 0.9$ in [0, 0, -η], and over a small energy range, $0.65 \leq E \leq 0.75$ meV, for $YbMgGaO_4$ at 35 K and 0 T using the incident neutron energy 5.5 meV.

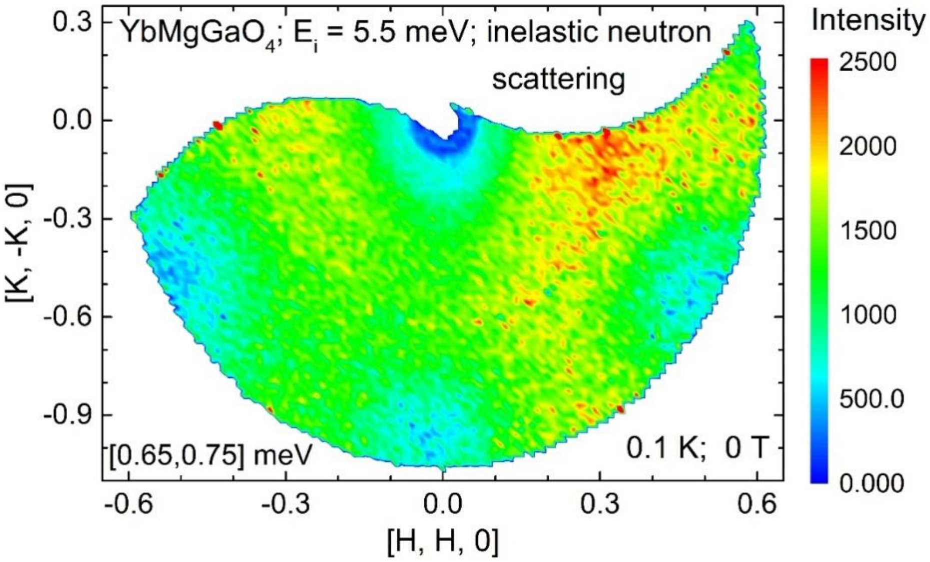

Supplementary Figure 8. Raw inelastic neutron scattering spectrum. The data have been integrated over the momentum space, -0.9 ≤ η ≤ 0.9 in [0, 0, -η], and over a small energy range, 0.65 ≤ E ≤ 0.75 meV, for $YbMgGaO_4$ at 0.1 K and 0 T using the incident neutron energy 5.5 meV.

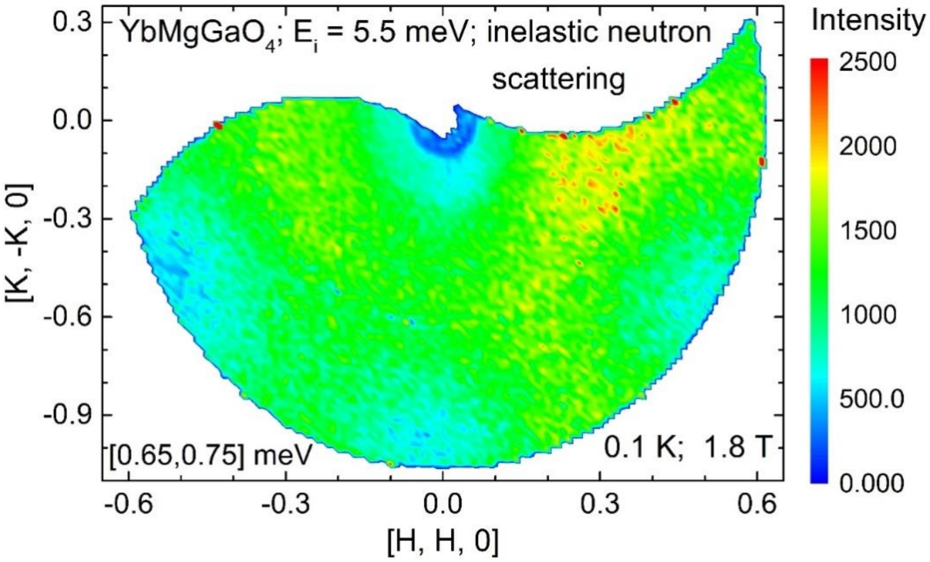

Supplementary Figure 9. Raw inelastic neutron scattering spectrum. The data have been integrated over the momentum space, -0.9 ≤ η ≤ 0.9 in [0, 0, -η], and over a small energy range, 0.65 ≤ E ≤ 0.75 meV, for $YbMgGaO_4$ at 0.1 K and 1.8 T using the incident neutron energy 5.5 meV.

Supplementary Note 2. Calculated equal-time INS intensity

In Fig. 1f of the main text, as well as in Fig. 1a and b, the coordinate point (x, y) represents the wave-vector, x[1, 1, 0] + y[1, -1, 0] = [x+y, x-y, 0] = $(x+y)\boldsymbol{\beta}_1+(x-y)\boldsymbol{\beta}_2+0\boldsymbol{\beta}_3$ (here, $\boldsymbol{\beta}_1$, $\boldsymbol{\beta}_2$ and $\boldsymbol{\beta}_3$ are the inverted lattice vectors). That is, $H = x+y$ and $K = x-y$ in Eq. 2 (see the main text).

The similar representation is used in other recent publications, such as Ref. 4 (triangular lattice) and Ref. 3 (kagome lattice). The corresponding equal-time INS intensity of uncorrelated nearest-neighbor valence bonds on a kagome lattice is proportional to,

$$|F(\mathbf{Q})|^2 = \frac{2}{3}|f(\mathbf{Q})|^2\{3 - cos(\pi H) - cos(\pi K) - cos[\pi(H + K)]\}. \quad (2)$$

For herbertsmithite, $f(\mathbf{Q})$ stands for the magnetic form factor of free Cu^{2+}, Supplementary Eq. 2 is exactly consistent with the calculations previously reported in the Ref. 3.

The equal-time INS intensity for uncorrelated next-nearest-neighbor valence bonds on the triangular lattice is proportional to,

$$|F(\mathbf{Q})|^2 = \frac{2}{3}|f(\mathbf{Q})|^2\{3 - cos[2\pi(2H + K)] - cos[2\pi(H + 2K)] - cos[2\pi(H - K)]\}. \quad (3)$$

The equal-time INS intensity for uncorrelated third-nearest-neighbor valence bonds on the triangular lattice is proportional to,

$$|F(\mathbf{Q})|^2 = \frac{2}{3}|f(\mathbf{Q})|^2\{3 - cos(4\pi H) - cos(4\pi K) - cos[4\pi(H + K)]\}. \quad (4)$$

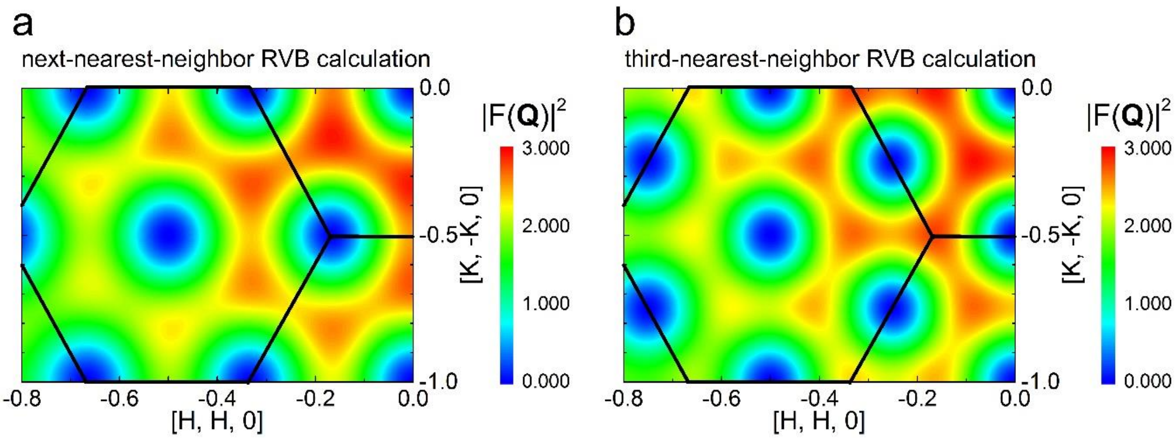

Supplementary Figure 10. Calculated equal-time scattering intensity. a, calculated inelastic neutron scattering intensity for uncorrelated next-nearest-neighbor valence bonds (Supplementary Eq. 3), and, **b**, for uncorrelated third-nearest-neighbor valence bonds (Supplementary Eq. 4). The black lines represent Brillouin zone boundaries. Both calculations are clearly very different from our experimental observations (Supplementary Fig. 7 and Fig. 8).

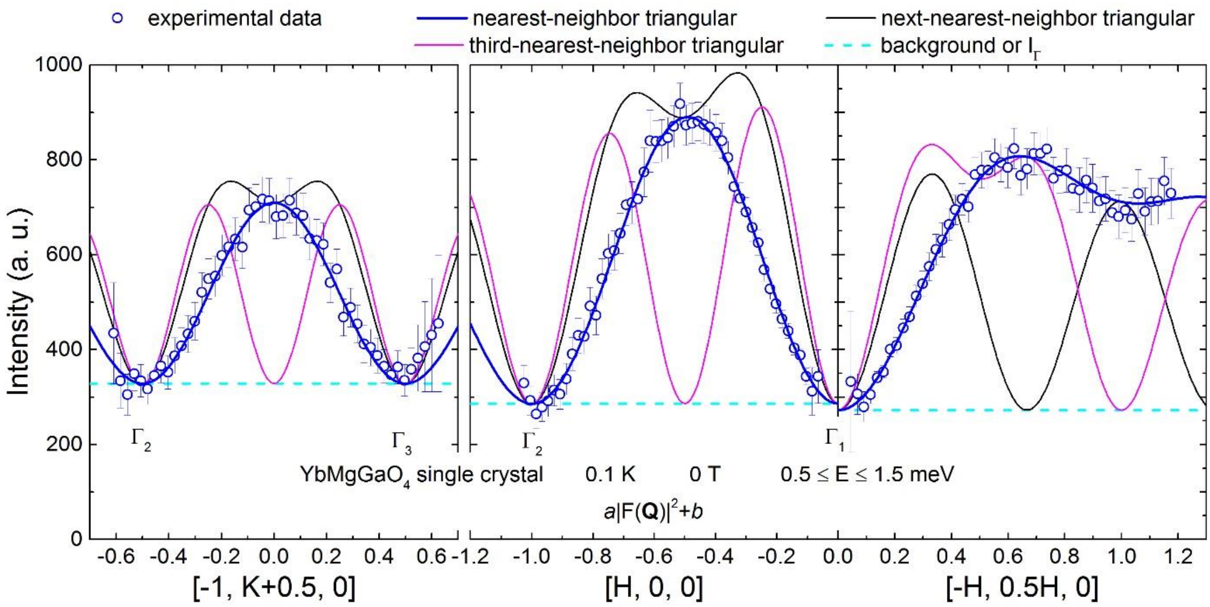

Supplementary Figure 11. Wave-vector dependence of the INS intensity. The data were measured along [-1, *K*+0.5, 0], [*H*, 0, 0], and [-*H*, 0.5*H*, 0], with lines representing different RVB calculations, $a|F(\mathbf{Q})|^2+b$. Here, *a* and *b* are fitting parameters, the pre-factor and background constant, respectively. Error bars on INS data indicate one standard error propagated from neutron counts (using Horace-Matlab). The **Q**-dependence of the signal follows the nearest-neighbor RVB model on the triangular lattice very well, while it is inconsistent with other valence bond correlations. Please note: Due to the unequal number of integrated data points, both *a* and *b* are slightly different along different **Q** directions. And there are slight boundary effects at the beginning and end of each series of the experimental data.

Supplementary Table 1. **Fitted pre-factors *a* and background constants *b*.** The fitted constants were obtained at 35 and 0.1 K using the nearest-neighbor RVB model on the triangular lattice.

Directions	[-1, *K*+0.5, 0] (Fig.1c)		[*H*, 0, 0] (Fig.1d)		[-*H*, 0.5*H*, 0] (Fig.1e)	
Temperature	35 K	0.1 K	35 K	0.1 K	35 K	0.1 K
(2/3)*a*	29.1(1.7)	109.6(2.7)	50.9(2.2)	158.3(4.6)	42.5(2.1)	126.4(3.9)
a(35K)/*a*(0.1K)	0.266(22)		0.322(23)		0.336(27)	
b or I_Γ	165.4(3.2)	328.6(4.9)	130.7(4.3)	286.1(8.5)	118.6(5.1)	272.5(9.3)
b(35K)/*b*(0.1K) or I_Γ(35K)/I_Γ(0.1K)	0.503(17)		0.457(29)		0.435(34)	

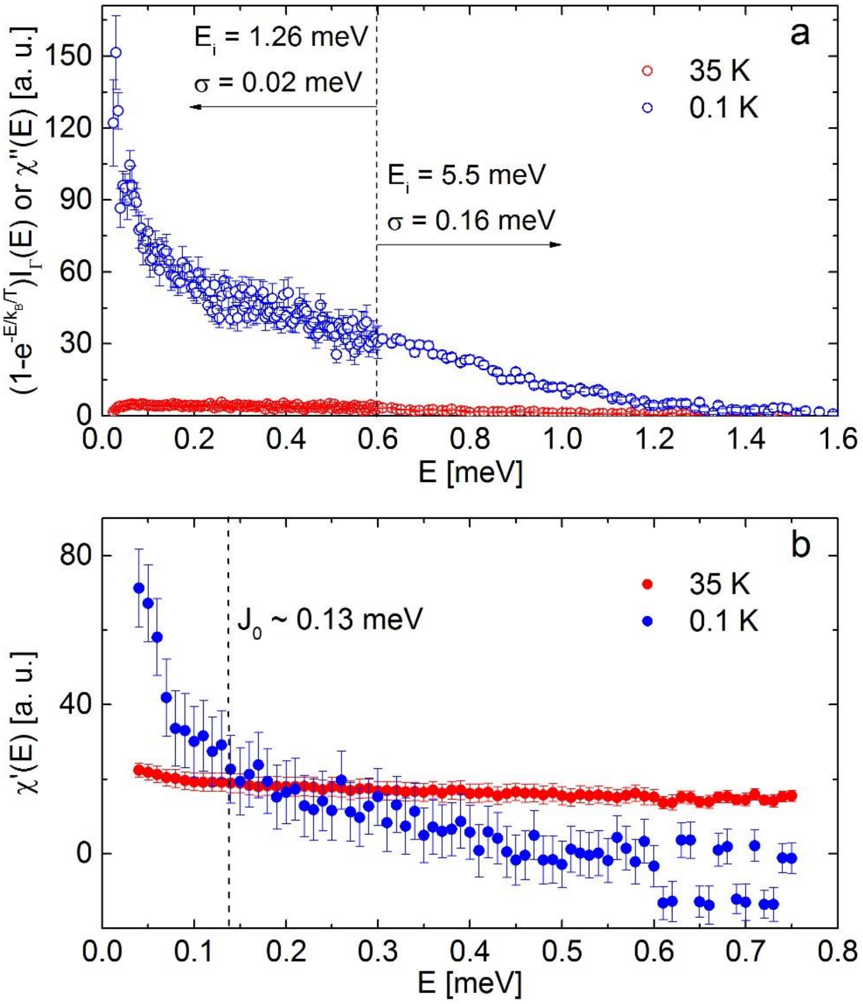

Supplementary Figure 12. Energy dependence of the uniform susceptibility. The data (**Q** ~ 0) were measured at 0.1 and 35 K. Error bars on susceptibility data indicate one standard error propagated from neutron counts. **a**, imaginary part of the susceptibility; **b** real part of the susceptibility. The elastic signal determined by fitting the spectrum at $|E| \leq \sigma$ was subtracted from $I_\Gamma(E)$. The spectra at $0.02 < E < 0.6$ meV were obtained from the measurements with E_i = 1.26 meV (σ = 0.02 meV), while the spectra at $E >$ 0.6 meV were obtained from the measurements with E_i = 5.5 meV (σ = 0.16 meV). The integration in the Kramers-Kronig relation was performed starting from 0.02 meV, and the data points with $|E'-E| <$ 0.005 meV were excluded in the integral to avoid the divergence (Supplementary Eq. 6).

Supplementary Note 3. Uniform spin susceptibility

At the Gamma point (Γ_1: $K = 0$; $H = 0$, and $\mathbf{Q} = 0$), the measured INS intensity, $I_\Gamma(E)$, is related to the uniform spin susceptibility, $\chi'(E)$. Via the fluctuation-dissipation theorem, we obtain imaginary part of the susceptibility[5],

$$\chi''(E) \propto \left(1 - e^{-\frac{E}{k_B T}}\right) I_\Gamma(E), \qquad (5)$$

assuming the Debye-Waller factor is temperature-independent below ~ 40 K.

Real part of the susceptibility can be obtained through the Kramers-Kronig transformation[5],

$$\chi'(E) \propto \frac{1}{\pi} \int \frac{\left(1 - e^{-\frac{E'}{k_B T}}\right) I_\Gamma(E')}{E' - E} dE'. \qquad (6)$$

Energy dependence of the uniform (**Q** ~ 0) susceptibility is shown in Supplementary Fig. 12. Experimental INS intensity (I_Γ) at 0.1 K is significantly larger than that at 35 K, I_Γ(0.1 K) ~ 2I_Γ(35 K) (0.5 ≤ E ≤ 1.5 meV). Nevertheless, above 0.5 meV real part of the susceptibility (χ') measured at 0.1 K turns out to be zero within the error bar, which is well in line with our RVB scenario. High values of χ' are seen at energies below ~ 0.15 meV only, indicating some low-energy physics beyond the simple RVB picture.

The comparison of χ' with the bulk susceptibility (Supplementary Fig. 13) indicates that the susceptibility at 0.48 K is higher than the one expected at 0.1 K, even at low energies, where $\chi'(E)$ is finite. Therefore, we anticipate a non-upward trend in the susceptibility around 0.1 K and exclude any ferromagnetic instability in the system.

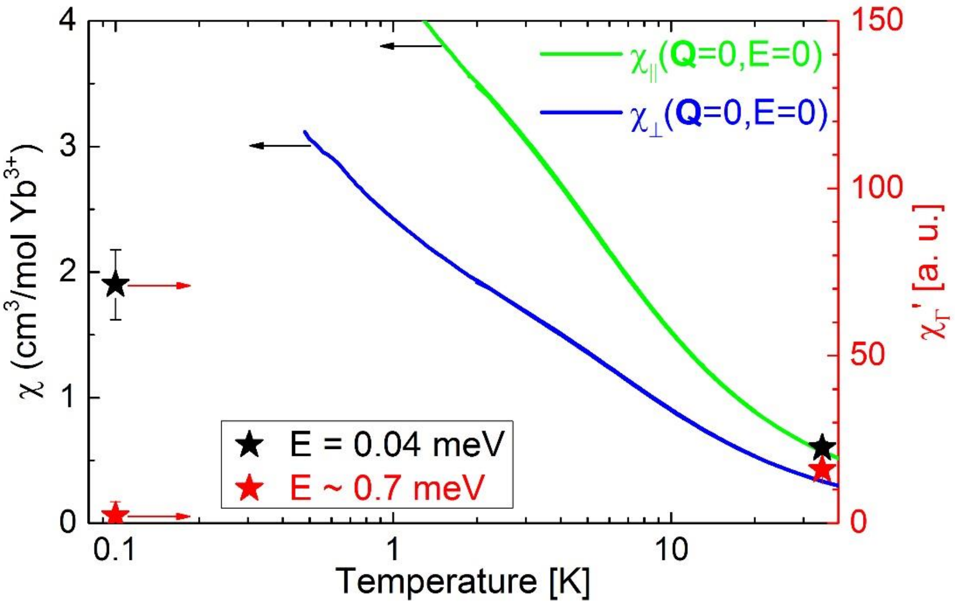

Supplementary Figure 13. Temperature dependences of the static bulk susceptibilities. The data were measured with the field parallel ($\chi_{||}$) and perpendicular ($\chi_\perp$) to the c-axis for the $YbMgGaO_4$ single crystal[6]. The black stars show the uniform spin susceptibilities at E = 0.04 meV, while the red stars show the uniform spin susceptibilities around E = 0.7 meV, as extracted from the INS data. Error bars on susceptibility data indicate one standard error propagated from neutron counts.

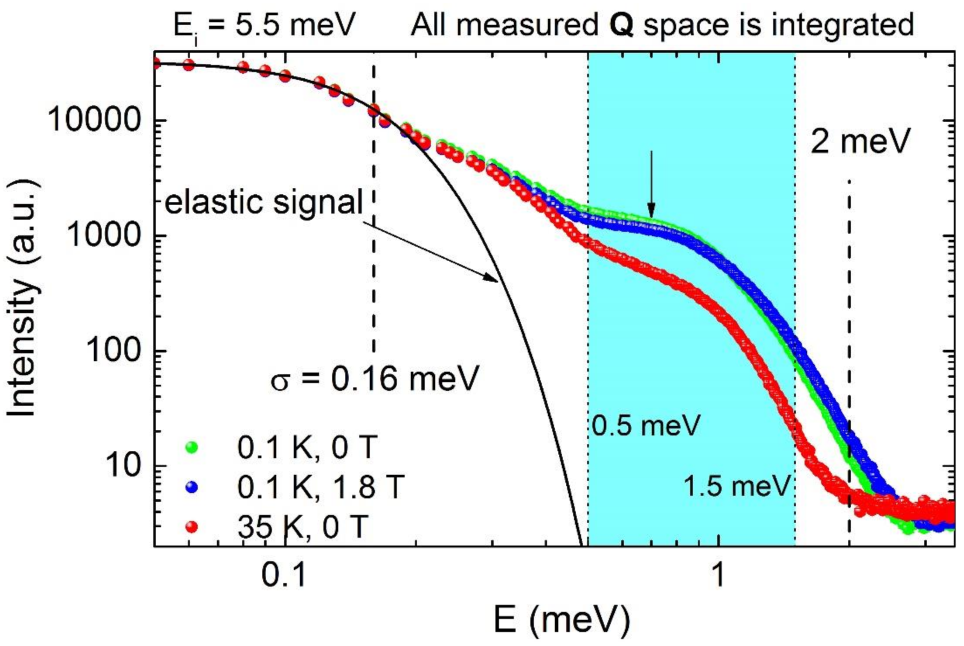

Supplementary Figure 14. Energy dependence of the neutron scattering intensity. The data were collected for $YbMgGaO_4$ with E_i = 5.5 meV. Solid line represents the elastic contribution. Error bars on INS data indicate one standard error propagated from neutron counts (using Horace-Matlab).

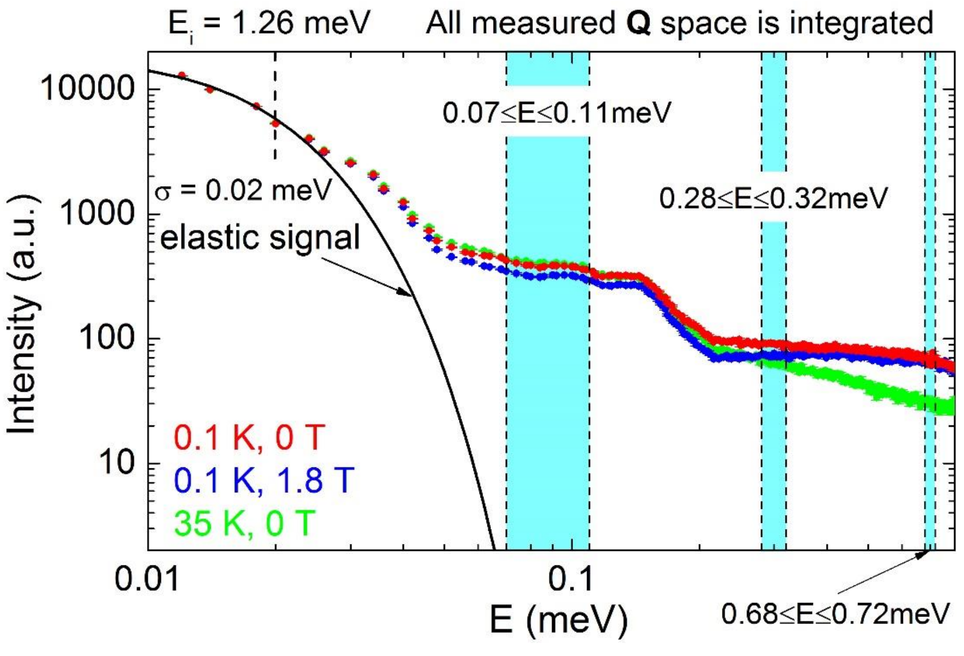

Supplementary Figure 15. Energy dependence of the neutron scattering intensity. The data (E_i = 1.26 meV) were collected with solid line showing the elastic component. Error bars on INS data indicate one standard error propagated from neutron counts (using Horace-Matlab).

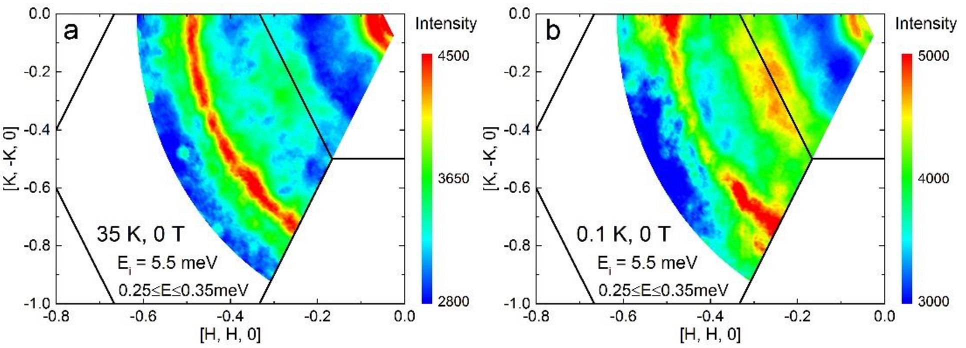

Supplementary Figure 16. Wave-vector dependence of the INS intensity. The **Q**-dependent INS data ($0.25 \leq E \leq 0.35$ meV, with $E_i = 5.5$ meV) were measured **a**, at 35 K and 0 T, **b**, at 0.1 K and 0 T. They are contaminated by a polycrystalline-like signal of some sort. It may originate from the magnet, cryostat, and/or sample holder.

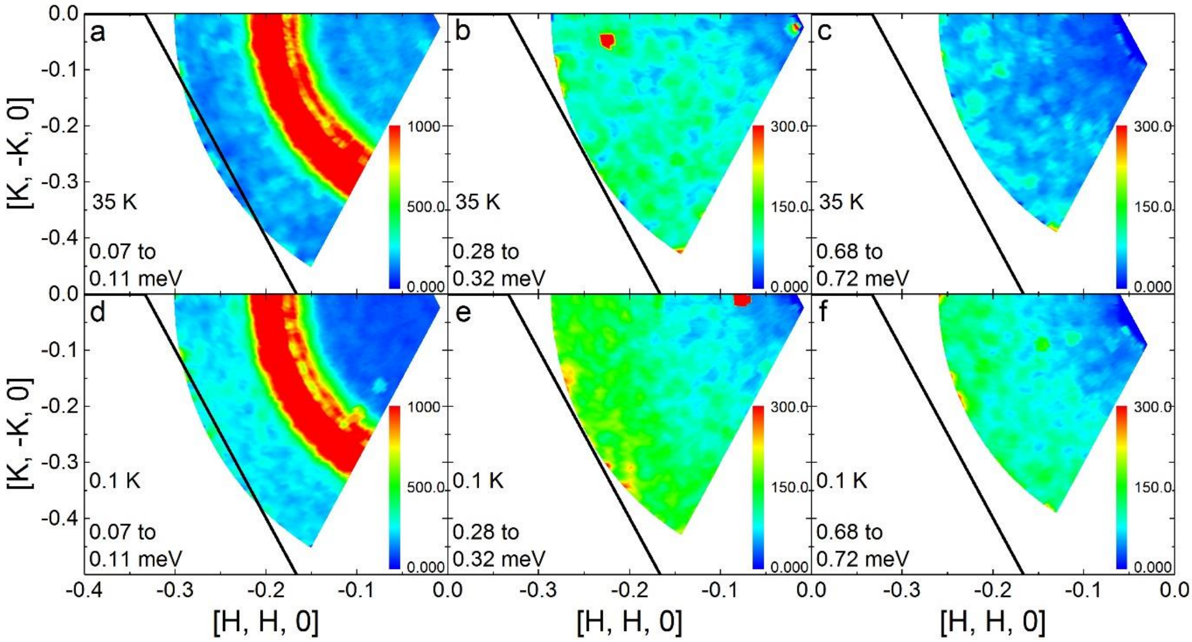

Supplementary Figure 17. Wave-vector dependence of the INS intensity. The **Q**-dependent INS data were measured under 0 T with $E_i = 1.26$ meV **a**, at $0.07 \leq E \leq 0.11$ meV and 35 K, **b**, at $0.28 \leq E \leq 0.32$ meV and 35 K, **c**, at $0.68 \leq E \leq 0.72$ meV and 35 K, **d**, at $0.07 \leq E \leq 0.11$ meV and 0.1 K, **e**, at $0.28 \leq E \leq 0.32$ meV and 0.1 K, and **f**, at $0.68 \leq E \leq 0.72$ meV and 0.1 K. There are no qualitative differences between the spectra at ~ 0.3 and ~ 0.7 meV in all measured **Q** space apart from an increase in the intensity (please compare b with c at 35 K and e with f at 0.1 K).

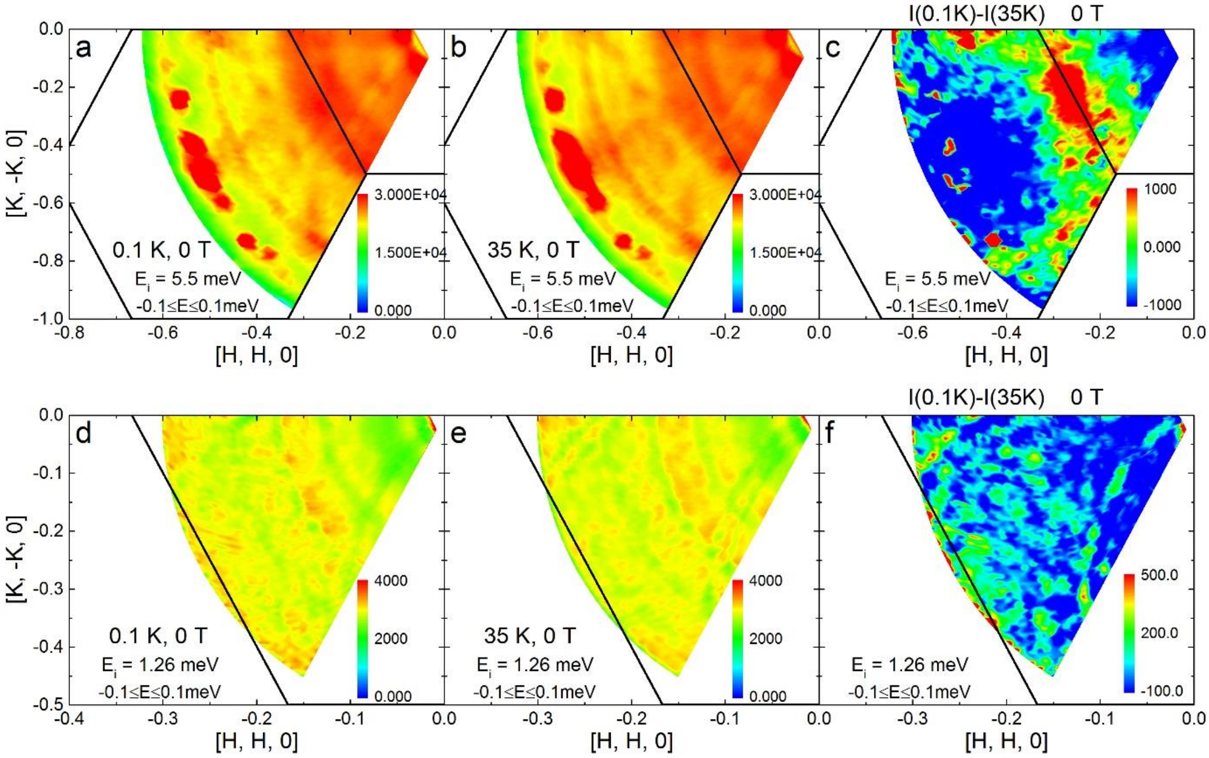

Supplementary Figure 18. Wave-vector dependence of the elastic scattering signal. The data were integrated over $-0.1 \leq E \leq 0.1$ meV: **a**. with $E_i = 5.5$ meV at 0.1 K, **b**. with $E_i = 5.5$ meV at 35 K, **d**. with E_i =1.26 meV at 0.1 K, and **e**. with $E_i = 1.26$ meV at 35 K. I(0.1 K) - I(35 K) **c**. with $E_i = 5.5$ meV, and **f**. with $E_i = 1.26$ meV. The polycrystalline signal of some sort seriously contaminates the intrinsic signal of $YbMgGaO_4$. We can subtract the 0.1 K signal with the 35 K signal, but the subtracted signals become significantly negative and seem too noisy in some regions of the **Q** space (see c and f), and the measured **Q** space is also very limited with $E_i = 1.26$ meV (see f).

Supplementary Note 4. Low energy Q-dependent INS spectra

The positive signal in I(0.1K) - I(35K) at $|E| \leq 0.1$ meV around the M-point is likely intrinsic (Supplementary Fig. 18c) and seems largely consistent with the earlier INS data by Paddison *et al.*[7] who observed maxima of the diffuse scattering at the M-points, as opposed to the maxima at the K-points above 0.5 meV. This shift of the intensity reflects a fundamental change from the predominant nearest-neighbor correlations at high energies to long-range correlations that manifest themselves at very low energies only. Detailed analysis of these long-range correlations is hindered by the contamination of our low-energy data with elastic signals from the sample environment.

Whereas the full energy range starting from 0 meV is unfeasible for the analysis, we argue that the **Q**-dependence of the intensity obtained by integrating the data above 0.5 meV is sufficient for drawing robust conclusions on the nature of spin-spin correlations underlying the excitation continuum. First, our analysis is performed along the lines of the recent INS study of herbertsmithite, where lower-energy spectral weight was also excluded. Specifically, for herbertsmithite the spectra were integrated starting from $E_{min} = 1$ meV, which is *well above* the energy resolutions ($1/2\sigma$), 0.21 ($E_{min}/\sigma = 2.4$) and 0.08 meV ($E_{min}/\sigma = 6.3$) (half-width at half-maximum, please see METHODS SUMMARY in Ref. 3). In our case, we use the same strategy

and adopt E_{min}/σ = 0.5/0.16 = 3.1 to completely exclude the elastic signal (Supplementary Fig. 14).

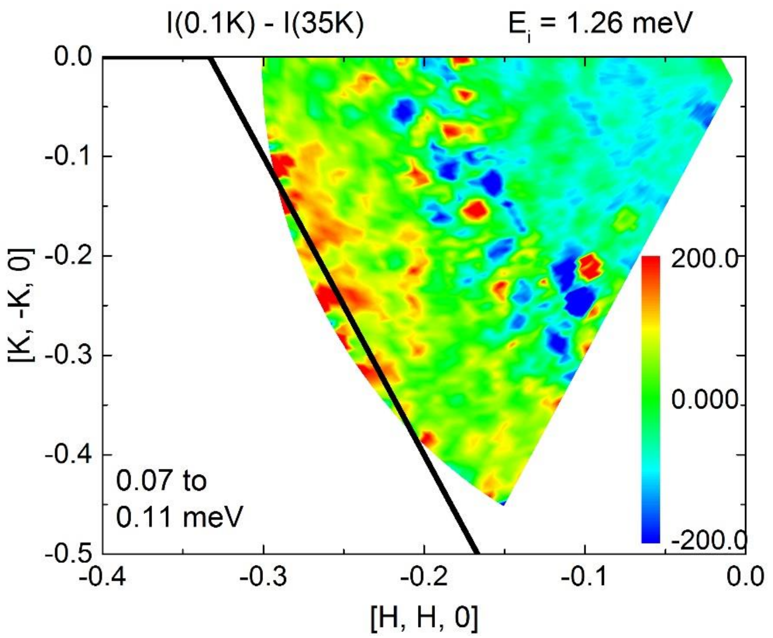

Supplementary Figure 19. Wave-vector dependences of the subtracted INS intensity. I(0.1K) – I(35K) were integrated over 0.07 ≤ E ≤ 0.11 meV with E_i = 1.26 meV. The subtracted signals become significantly negative in some regions of the **Q** space.

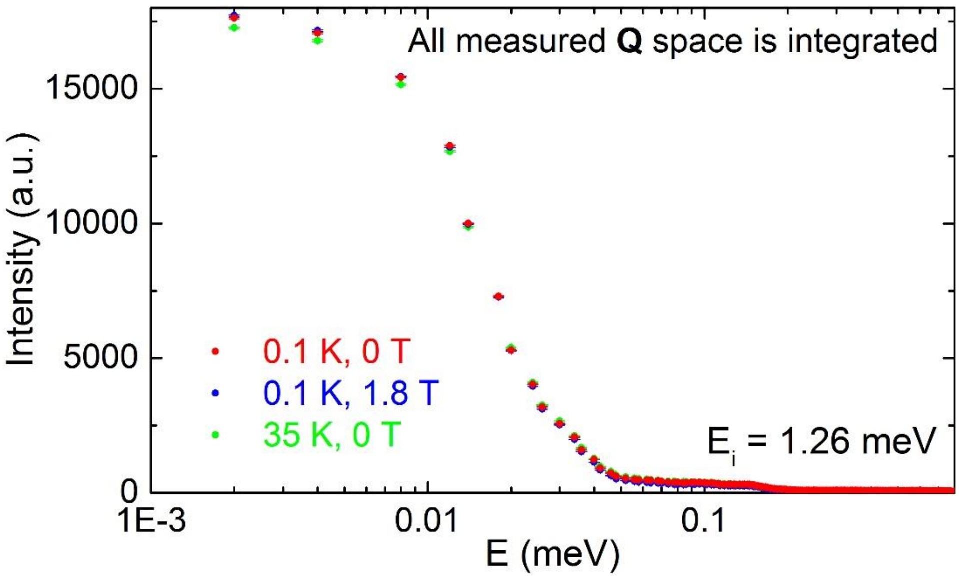

Supplementary Figure 20. Raw INS data for $YbMgGaO_4$. The data were collected with the lowest incident neutron energy of 1.26 meV. The data have been integrated over all measured **Q** space. Error bars on INS data indicate one standard error propagated from neutron counts (using Horace-Matlab).

Supplementary Note 5. Spin-wave excitations for the 120 degree magnetic structure

Further on, we verified that the spectral weight about 0.5 meV bears characteristic signatures of the ground state. To this end, we considered an alternative state, the 120-deg long-range order, and calculated spin-wave excitations using experimental parametrization of the spin Hamiltonian of $YbMgGaO_4$ (J_{zz} = 0.085 meV, $J_{\pm}$ = 0.078 meV, $J_{\pm\pm}$ = 0.013 meV and $J_{z\pm}$ = 0.003 meV)[2]. Magnetic excitations calculated on the level of the linear spin-wave theory[8] (Supplementary Fig. 21), were broadened with the energy resolution of the LET spectrometer, σ = 0.16 meV (Supplementary Fig. 22). We integrate the calculated spectrum from half the spin-wave bandwidth (~ 0.2 meV) to energies well above the top of the band (~ 0.5 meV) (Supplementary Fig. 22). The magenta curves in Supplementary Fig. 23 show that the profile of the integrated spin-wave spectrum is clearly inconsistent with the experimental one. For the sake of completeness, we also show the spin-wave spectra integrated from 0, 0.12, 0.3, 0.34 and 0.4 meV. None of them is qualitatively consistent with the experimental data (Supplementary Fig. 23).

A similar result can be obtained for the nearest-neighbor Heisenberg Hamiltonian on the triangular lattice that also yields the 120-deg long-range-ordered state. We thus conclude that even at high energies excitations from the 120-deg ordered state are distinct from those of the RVB state. Therefore, even above 0.5 meV (about 1/3 of the experimental bandwidth) the **Q**-dependence of the scattered intensity remains characteristic, and indicates predominant nearest-neighbor correlations, which are likely of the RVB type (see detailed discussion in the main text).

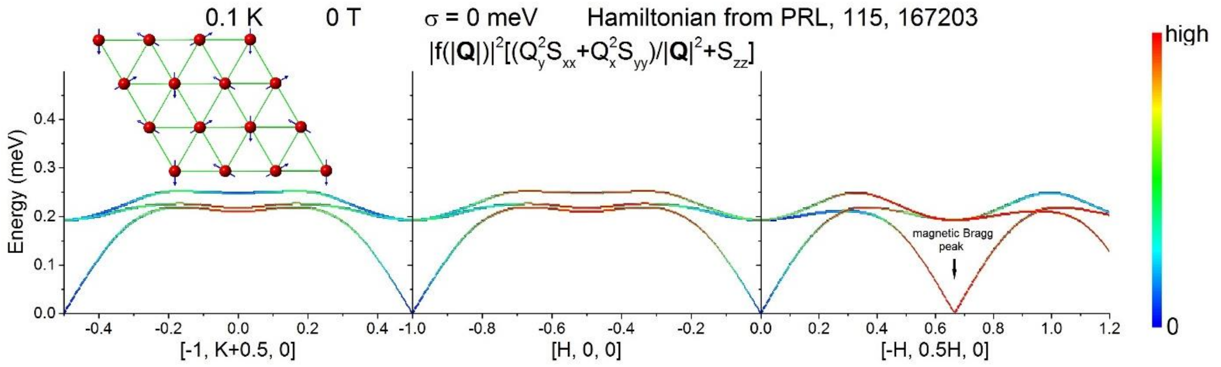

Supplementary Figure 21. Calculated spin-wave excitations. The data were calculated along [-1, *K*+0.5, 0], [*H*, 0, 0], and [-*H*, 0.5*H*, 0] for the hypothetical 120 degree magnetically ordered structure (see the inset for the magnetic unit cell) based on the experimental parametrization of the spin Hamiltonian of $YbMgGaO_4$[2,5]. Here, $f(|\mathbf{Q}|)$ is the magnetic form factor of Yb^{3+}.

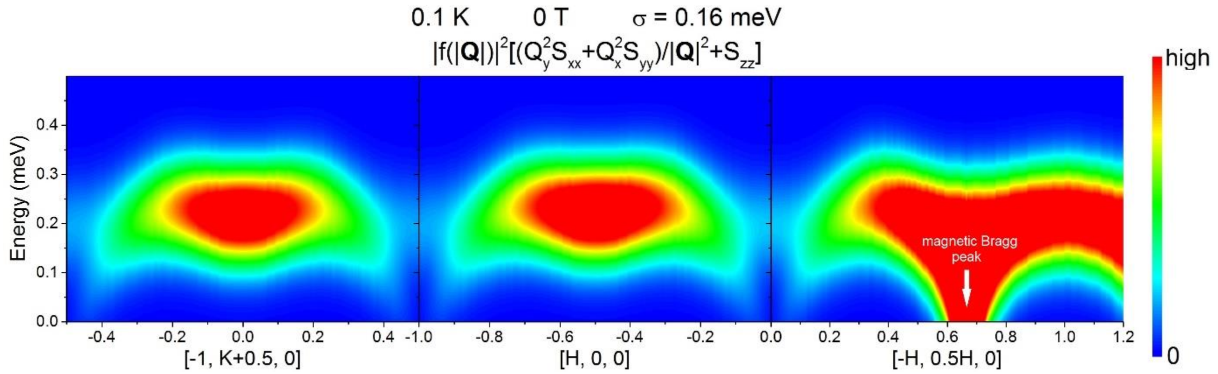

Supplementary Figure 22. Calculated spin-wave excitations. The calculation was based on the experimental parametrization of the spin Hamiltonian (Supplementary Fig. 21) with the Gaussian broadening, FWHM = 0.16 meV.

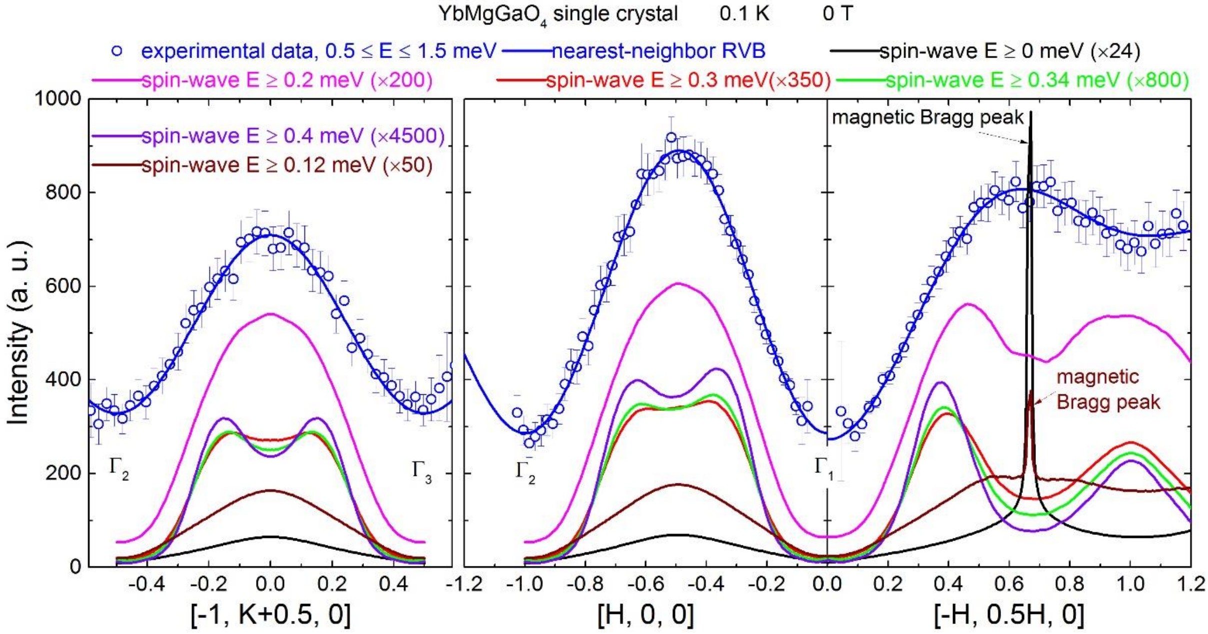

Supplementary Figure 23. Wave-vector dependence of the INS intensity. The INS data were collected along [-1, *K*+0.5, 0], [*H*, 0, 0], and [-*H*, 0.5*H*, 0], with blue lines representing nearest-neighbor RVB calculations, $a|F(\mathbf{Q})|^2+b$. The spin-wave spectra based on the experimental parametrization of the spin Hamiltonian are integrated from 0, 0.12, 0.2, 0.3, 0.34, and 0.4 meV (all up to 0.5 meV) yielding the black, wine, magenta, red, green and violet curves, respectively. Above 0.3 meV, only minor changes in the **Q**-dependence are observed showing that even at very high energies the **Q**-dependence of the RVB state is not reproduced. The magnetic Bragg peak (-2/3, 1/3, 0), which is naturally present in the spin-wave spectra, reflects long-range order in the 120-deg state and disappears at high energies. Error bars on INS data indicate one standard error propagated from neutron counts (using Horace-Matlab).

The calculated spin-wave excitations based on the Heisenberg model without any broadening are shown in Supplementary Fig. 24. With an arbitrary Gaussian broadening, FWHM ~ 2*J*, the magnetic Bragg reflection (-2/3, 1/3, 0) of the periodic magnetic structure based on the Heisenberg model is very clear and is much stronger than that based on the $YbMgGaO_4$ Hamiltonian (Supplementary Fig. 25). To exclude this sharp Bragg peak reflecting the long-range correlations, we obtain the high-energy spin-wave excitations by integrating from 3.4*J* to 4*J* (Supplementary Fig. 26).

Both the calculated high-energy spin-wave spectra for the same magnetic structure indeed share almost the same profile, though the corresponding spin Hamiltonians are completely symmetrically different (see the pink and violet curves in Supplementary Fig. 26). Both calculated high-energy spectra without magnetic Bragg reflections only reflect the short distance spin correlations of the 120 degree Neel state, while are essentially inconsistent with our measured high-energy INS spectrum (Supplementary Fig. 26).

The **Q**-dependence of the measured high-energy INS spectrum for $YbMgGaO_4$ cannot be understood by whatever short distance antiferromagnetic spin correlations on a triangular lattice, and the nearest-neighbor RVB scenario is still the most accurate explanation so far.

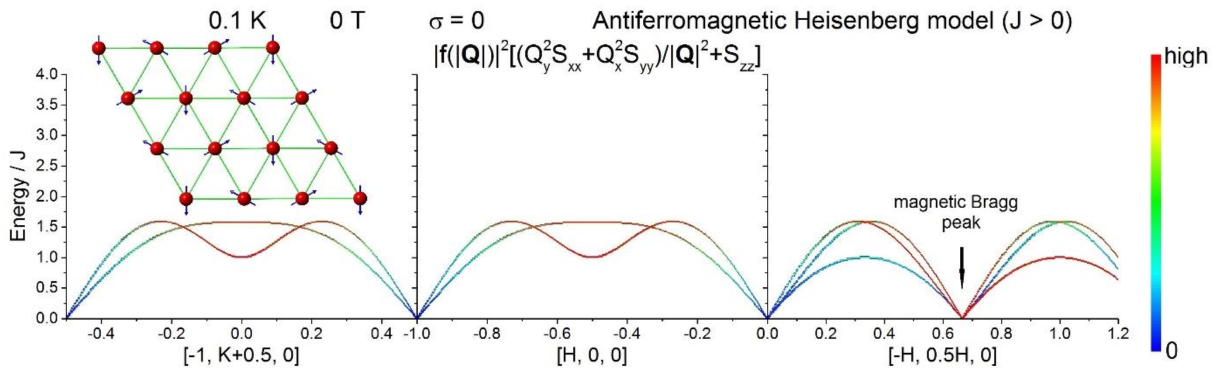

Supplementary Figure 24. Calculated spin-wave excitations. The data were calculated along [-1, *K*+0.5, 0], [*H*, 0, 0], and [-*H*, 0.5*H*, 0] for the 120 degree magnetically ordered ground state (see the inset for the magnetic unit cell) based on the nearest-neighbor spin-1/2 antiferromagnetic Heisenberg Hamiltonian on the triangular lattice ($J > 0$).

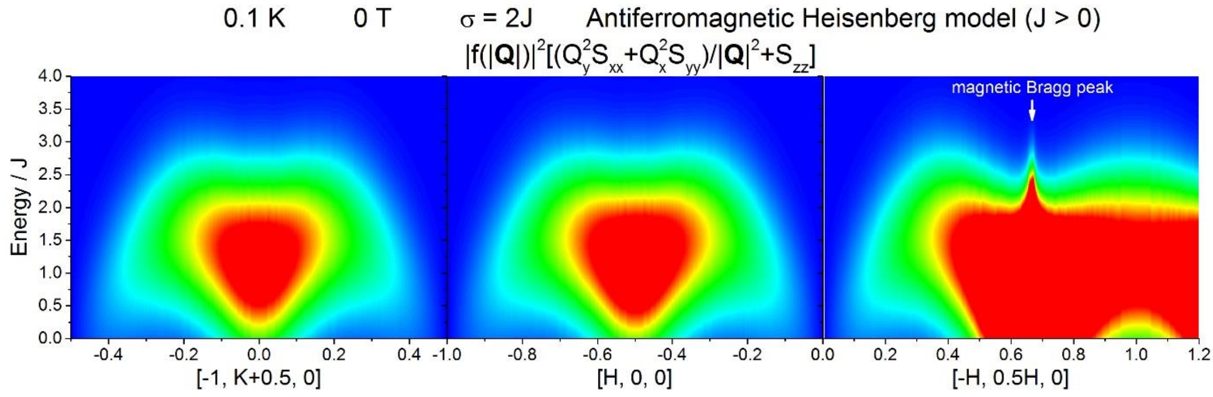

Supplementary Figure 25. Calculated spin-wave excitations. The data were calculated with the Gaussian broadening, FWHM = $2J$ (Supplementary Fig. 24).

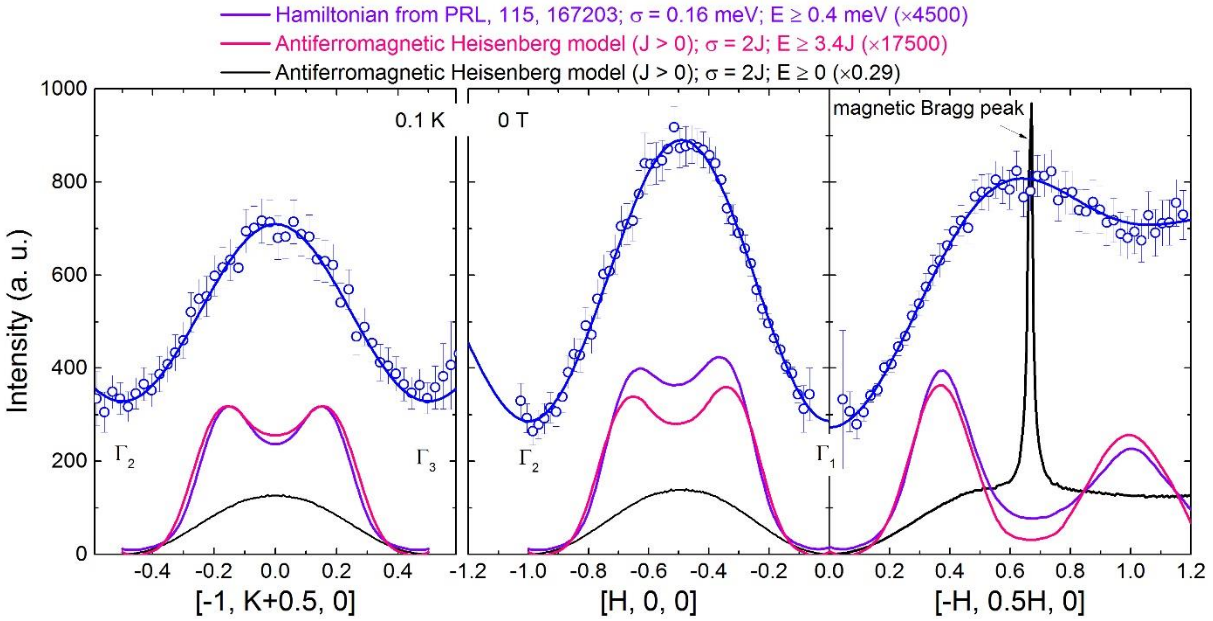

Supplementary Figure 26. Wave-vector dependence of the INS intensity. The INS data were collected along [-1, K+0.5, 0], [H, 0, 0], and [-H, 0.5H, 0], with blue lines representing nearest-neighbor RVB calculations, $a|F(\mathbf{Q})|^2+b$. The spin-wave spectra for the nearest-neighbor Heisenberg Hamiltonian are integrated from 0J and 3.4J (both up to 4J) yielding the black and pink curves, respectively. The violet curves show the integration result for spin-wave excitations calculated for the $YbMgGaO_4$ Hamiltonian (the violet curves in Supplementary Fig. 23). Error bars on INS data indicate one standard error propagated from neutron counts (using Horace-Matlab).

Supplementary References

1. Li, Y. S. *et al.* Crystalline electric-field randomness in the triangular lattice spin-liquid $YbMgGaO_4$. *Phys. Rev. Lett.* **118**, 107202 (2017).

2. Li, Y. S. *et al.* Rare-earth triangular lattice spin liquid: a single-crystal study of $YbMgGaO_4$. *Phys. Rev. Lett.* **115**, 167203 (2015).

3. Han, T. H. *et al.* Fractionalized excitations in the spin-liquid state of a kagome-lattice antiferromagnet. *Nature* **492**, 406-410 (2012).

4. Shen, Y. *et al.* Evidence for a spinon Fermi surface in a triangular-lattice quantum-spin-liquid candidate. *Nature* **540**, 559-562 (2016).

5. Xu, G., Xu, Z. & Tranquada, J. M. Absolute cross-section normalization of magnetic neutron scattering data. *Rev. Sci. Instrum.* **84**, 083906 (2013).

6. Li, Y. S. *et al.* Muon spin relaxation evidence for the U(1) quantum spin-liquid ground state in the triangular antiferromagnet $YbMgGaO_4$. *Phys. Rev. Lett.* **117**, 097201 (2016).

7. Paddison, J. A. M. *et al.* Continuous excitations of the triangular-lattice quantum spin liquid $YbMgGaO_4$. *Nat. Phys.* **13**, 117-122 (2017).

8. Toth, S. & Lake, B. Linear spin wave theory for single-Q incommensurate magnetic structures. *J. Phys.: Condens. Matter* **27**, 166002 (2015).
